# Protein quality in ready‐to‐use supplementary foods for moderate wasting

**DOI:** 10.1111/mcn.13019

**Published:** 2020-05-19

**Authors:** Rebecca Roediger, Hans‐Henrik Stein, Meghan Callaghan‐Gillespie, Jeffrey Kahn Blackman, Kristin Kohlmann, Kenneth Maleta, Mark Manary

**Affiliations:** ^1^ Division of Gastroenterology, Department of Medicine Washington University St. Louis Missouri USA; ^2^ Department of Animal Science University of Illinois Urbana Illinois USA; ^3^ Department of Pediatrics Washington University St. Louis Missouri USA; ^4^ Department of Community Health, College of Medicine University of Malawi Blantyre Malawi

**Keywords:** dairy products, indispensable amino acids, Malawi, moderate acute malnutrition, protein quality, ready‐to‐use supplementary food, wasting

## Abstract

There are no guidelines for the optimal protein quality of ready‐to‐supplementary food (RUSF) for moderate acute malnutrition (MAM). This randomized, controlled, double‐blinded, clinical effectiveness trial evaluated two RUSFs in the treatment of MAM. Both foods contained greater than 7% dairy protein, but the protein‐optimized RUSF had a calculated digestible indispensable amino acid score (DIAAS) of 95%, whereas the control RUSF had a calculated DIAAS of 63%. There were 1,737 rural Malawian children 6–59 months of age treated with 75 kcal/kg/day of either control or protein quality‐optimized RUSF for up to 12 weeks. There was no difference in the proportion of children who recovered from MAM between the group that received protein‐optimized RUSF (759/860, 88%) and the group that received control RUSF (766/877, 87%, difference 1%, 95% CI, −2.1 to 4.1, *p* = 0.61). There were no differences in time to recovery or average weight gain; nor were adverse effects reported. Both RUSFs showed indistinguishable clinical outcomes, with recovery rates higher than typically seen in treatment for MAM. The DIAAS of these two RUSFs was measured using a pig model. Unexpectedly, the protein quality of the optimized RUSF was inferior to the control RUSF: DIAAS = 82% for the protein quality optimized RUSF and 96% for control RUSF. The controlled conditions of this trial suggest that in supplementary food products for MAM, protein quality is not an independent predictor of clinical effectiveness.

**Key messages**

- This randomized, controlled, double‐blind, clinical effectiveness trial compared two ready‐to‐use supplementary foods of differing protein quality in treatment of moderate acute malnutrition (MAM) in Malawi.
- Although the protein quality scores of the two foods differed, there was no difference in the rate of recovery from MAM, rate of weight gain, time to recovery or average mid‐upper arm circumference or weight gain between the groups.
- Both foods had ample amounts of dairy protein and delivered higher rates of recovery from MAM than seen in most feeding trials.
- Protein quality scores were not associated with clinical outcomes in treatment of MAM.

## 1 INTRODUCTION

The annual incidence of moderate acute malnutrition (MAM) is 15% of all children in sub‐Saharan Africa (Marzia, Laura, & Paola, 2013). Children are especially vulnerable in their first few years of life when their energy demands are highest (Ghosh, 2016). Impairments of their immunity from MAM make children susceptible to infectious insults, which further compromises their nutritional status. Reduced cognition from MAM may lead to chronic deficits, resulting in a lifetime of decreased productivity and economic earnings, which contribute to the cycle of poverty. Up to 10.2% of infant mortality can be attributed to MAM (Marzia et al., 2013).

Although there is no widely implemented management protocol for MAM, several supplementary food products, including lipid based ready‐to‐use supplementary foods (RUSFs), have been formulated and successfully used. RUSF has been shown to increase recovery rates and decrease time to recovery when compared with corn soy blends (Suri, Moorthy, & Rosenberg, 2016).

The optimal protein composition of RUSF is uncertain. Quantifying only the total protein content in supplemental feeding can overestimate the amount of utilizable protein (Ghosh, 2013). Protein quality is a measurement that aims to quantify the capacity of a food to meet one's essential amino acid (AA) requirements based on physiologic status and body size. The quality of a protein is determined by assessing its essential AA composition as well as the digestibility of its AAs (FAO Expert Consultation, 2013; Food and Agriculture Organization of the United Nations, 2017).

Protein and AA content in supplementary food products is vital, because the recipients receiving supplementary foods often have no or limited access to high‐quality protein and different physiologic needs than a healthy population (Briend et al., 2015; Manary & Callaghan, 2017). Studies have shown the addition of high‐quality proteins, such as dairy proteins, to supplementary foods results in higher recovery rates and improved growth when compared with foods with only plant protein sources (Fabiansen et al., 2017; Schlossman et al., 2017; Stobaugh et al., 2016). One study found that a whey RUSF led to better rates of recovery than a soy protein RUSF despite the whey RUSF protein providing 33% less overall total protein content (Stobaugh et al., 2016). It is not known what component of whey RUSF accounted for the better recovery rates, whether it was attributable to the higher protein quality, the presence of bioactive peptides, dairy's action as a prebiotic or a combination of these. Regulating agencies are turning to protein quality metrics in their guidelines to standardize RUSF composition. However, gaps still remain for the optimal quality, quantity and source of protein to be included in supplementary foods (Eaton et al., 2019; Scherbaum & Srour, 2018), and to our knowledge, no prior clinical trial has compared two RUSFs with differing protein quality but both with high amounts of dairy protein.

The aim of this clinical trial was to compare the effectiveness of a protein quality‐optimized RUSF (HiPro‐RUSF) with an isonitrogenous control RUSF (C‐RUSF) in the treatment of MAM. The hypothesis was that recovery from MAM of children receiving HiPro‐RUSF would be superior to that for children receiving the C‐RUSF.

## 2 METHODS

### 2.1 Study foods

Subjects received about 75 kcal/kg/day of one of two types of RUSF. The RUSFs were isoenergetic and isonitrogenous, but HiPro‐RUSF had a higher calculated protein quality than the C‐RUSF. Both RUSFs were peanut based with inclusion of various dairy components. HiPro‐RUSF contained non‐fat dried skim milk and whey permeate, whereas C‐RUSF contained whey permeate and whey protein concentrate (WPC) (Table 1). Micronutrient composition of HiPro‐RUSF and C‐RUSF was similar (Table S1). Both RUSFs provided the FAO‐recommended amount of each essential AA for healthy 1‐ to 2‐year‐olds (Table 2). We estimated the AA requirements for catch‐up growth of 5 g/kg/day, using values provided by FAO for 10 g/kg/day for severe acute malnutrition (SAM) and using factorial approach to calculate the requirements for 5 g/kg/day catch‐up growth to better reflect the goals of treatment for MAM (Food and Agriculture Organization of the United Nations, 2017; Food and Nutrition Board, 2005; Joint WHO/FAO/UNU Expert Consultation, 2007). Our calculations indicated that with the exception of histidine in C‐RUSF, the RUSFs provided enough of each essential AA to achieve this catch‐up growth.

**TABLE 1:** Ingredient and macronutrient composition of two RUSFs used to treat moderate malnutrition

	Protein quality‐optimized RUSF	Control RUSF	
**Ingredient**			
Peanut paste g/100 g	10	19	
Sugar g/100 g	17	23	
Extruded soy flour g/100 g	4	‐	
Non‐fat dried skim milk g/100 g	23.8	‐	
Whey permeate g/100 g	15	23.50	
Whey protein concentrate (WPC80) g/100 g	‐	8.7	
Palm oil g/100 g	7.62	2.36	
Canola oil g/100 g	18.08	18.94	
Micronutrient mixture g/100 g	3.5	3.5	
Hydrogenated vegetable oil containing ~40% monoacylglycerides g/100 g	1.0	1.5	
**Macronutrients**			Recommended values^a^
Energy kcal/100 g	530	537	510
Protein g/100 g	13.46	13.42	11
Dairy protein g/100 g	8.72	7.20	5.5
Lactose g/100 g	25.83	20.50	‐
Total lipids g/100 g	32.8	32.9	26
**Essential amino acid**			Reference pattern for catch‐up Growth^b^
Histidine mg/g	24.49	15.28	24
Isoleucine mg/g	41.37	43.52	35
Leucine mg/g	80.86	73.77	74
Lysine mg/g	61.91	64.52	65
Threonine mg/g	35.43	44.16	36
Valine mg/g	49.48	42.78	46
Sulphur AA mg/g	29.63	37.45	31
Aromatic AA mg/g	78.60	71.99	63
Tryptophan	10.74	11.19	10

Abbreviations: AA, amino acid; RUSF, ready‐to‐use supplementary food.

a WFP Technical Specifications for RUSF 2019.

b FAO Report 2017: Reference pattern of amino acid content per gram of protein content in ready‐to‐use‐therapeutic foods for treatment of severe acute malnutrition.

**TABLE 2:** Amino acids provided by the RUSFs

Amino acid	Protein quality‐optimized RUSF mg/kg/day	Control RUSF mg/kg/day	Reference requirement mg/kg/day^a^	Requirement for 5 g/kg/day catch‐up growth mg/kg/day^b^
Histidine	48.16	30.05	15	39
Isoleucine	81.36	85.60	27	60
Leucine	159.03	145.08	54	123
Lysine	121.75	126.89	45	110
Threonine	69.68	86.84	23	61
Valine	97.32	84.14	36	81
Sulphur AAs	58.27	73.65	22	53
Aromatic AAs	154.59	141.58	40	104
Tryptophan	21.18	22.09	6.4	17

Abbreviations: AA, amino acid; RUSF, ready‐to‐use supplementary food.

a FAO Report 2017: Reference requirement of the daily amino acid amount needed per kilogram of a healthy 1‐ to 2 year‐old child for maintenance and normal growth.

b FAO Report 2017: Adapted from the reference requirement for daily amino acid amount needed per kilogram of a child with severe acute malnutrition for 10 g/kg/day of catch‐up growth. Adapted to 5 g/kg/day catch‐up growth to better reflect the goals of treatment in moderate acute malnutrition.

Both RUSFs were produced at Project Peanut Butter in Lunzu, Malawi, and underwent quality assurance and safety testing for aflatoxin and the presence of *Enterbacter spp* or *Salmonella* at the Malawi Bureau of Standards and Eurofins Scientific Inc., Des Moines, Iowa.

### 2.2 Protein quality calculations

Protein quality scores attempt to estimate the necessary protein composition of treatment foods. The FAO/WHO expert consultation on protein quality evaluation recommends two metrics for evaluating the protein quality of human foods: the protein digestibility‐corrected amino acid score (PDCAAS) and the digestible indispensable amino acid score (DIAAS) (Food and Agriculture Organization of the United Nations, 2017). Both of these methods apply a correction for digestibility to the indispensable AA content of a food or diet and relate it to a reference AA pattern. The DIAAS method uses a correction for digestibility based on the individual indispensable AA ileal digestibility measured from ileal sampling, whereas the PDCAAS method uses a single value of crude protein digestibility measured in faeces. The DIAAS approach is currently recommended over PDCAAS because the gut microbiota utilize and metabolize the AAs before they are excreted in the stool and obfuscate accurate absorption fractions based on faecal measurements used in the PDCAAS method. The nutrient needs of the individual are important when evaluating protein quality, as AA synthesis is affected by disease state and growth rate (Callaghan, Oyama, & Manary, 2017). AA deficiencies due to limited diet diversity are augmented by malabsorption due to environmental enteric dysfunction or diversion of AAs to mount an inflammatory response (Owino et al., 2016; Reeds, Fjeld, & Jahoor, 1994; Semba et al., 2016).

The calculated DIAAS score for the C‐RUSF using the FAO reference range for MAM was 63.5%, whereas for the HiPro‐RUSF, it was 94.9% (Table 3). The calculated PDCAAS score for the C‐RUSF for MAM was 74.6%, and it was 95.5% for the HiPro‐RUSF. The HiPro‐RUSF does not differ in protein quality between the PDCAAS and the DIAAS methods. However, the C‐RUSF has a significant drop in the DIAAS score as compared with the PDCAAS score.

**TABLE 3:** Calculated protein quality scores of RUSFs

Protein quality scoring method	Reference population^a^	Protein quality‐optimized RUSF	Control RUSF
PDCAAS	Moderate acute malnutrition	95.5%	74.6%
DIAAS	Moderate acute malnutrition	94.9%	63.5%
PDCAAS	Healthy 6‐month‐old	109.7%	89.5%
DIAAS	Healthy 6‐month‐old	108.3%	76.7%
PDCAAS	Healthy 1‐ to 2‐year‐old	118.5%	99.4%
DIAAS	Healthy 1‐ to 2‐year‐old	118.1%	85.2%

Abbreviations: DIAAS, digestible indispensable amino acid score; PDCAAS, protein digestibility‐corrected amino acid score; RUSF, ready‐to‐use supplementary food.

a Protein quality score was calculated on the basis of FAO 2017 reference pattern of required amino acid content per gram of protein necessary for the proper growth of the indicated population. Table 1 includes the reference pattern for the moderate acute malnutrition population.

Both DIAAS and PDCAAS rely on the most limited AA for their score. The DIAAS also accounts for the digestibility of the AAs. Table 1 shows the AA composition in milligrams per gram of protein of our RUSF alongside the AA reference pattern for MAM based on the FAO guidelines (Food and Agriculture Organization of the United Nations, 2017). This shows that the DIAAS score was determined by histidine for the C‐RUSF and lysine for the HiPro‐RUSF, as these are the least abundant digestible AAs compared with the reference pattern.

These calculations were based on the AA content and digestibility of our ingredients from CVB feed tables data (Blok & Spek, 2016). The calculations for whey permeate and WPC were based on data from Dairy Global Nutrition (U.S. Dairy Export Council, 2005) as the CVB feed tables did not contain data for these ingredients. However, there may be considerable variability in the protein content in WPC because it is produced from a by‐product of cheese processing. Depending on the type of cheese being created, the WPC protein content and AA composition will vary. Table 4 shows the measured AA composition of six WPCs as compared with our calculated WPC content for our C‐RUSF. When compared with our calculations based on WPC content from the Dairy for Global Nutrition data (U.S. Dairy Export Council, 2005), AAs in WPC can vary considerably. For example, histidine can be up to 43% higher and lysine content can be 17% less than what we used in our calculations (Table 4). Therefore, WPC calculations used to design our study may not necessarily reflect the WPC that was eventually used.

**TABLE 4:** Analysed nutrient composition of six whey protein concentrates based on as‐fed basis compared with calculated values used for designing C‐RUSF

Indispensable amino acids	WPC 80K (Arla) absolute percent (fraction of calculated WPC)	WPC MIA80 (Arla) absolute percent (fraction of calculated WPC)	WPC (Mathai, Liu, & Stein, 2017) absolute percent (fraction of calculated WPC)	WPC (Rutherfurd, Fanning, Miller & Moughan, 2014)^a^ absolute percent (fraction of calculated WPC)	WPC (NRC, 2012) absolute percent (fraction of calculated WPC)	WPC (Stein Feed Database) absolute percent (fraction of calculated WPC)	Calculated WPC C‐RUSF absolute percent
Arg	1.93 (0.97)	2.23 (1.12)	2.38 (1.19)	2.50 (1.25)	2.01 (1.01)	1.96 (0.98)	2.00
His	1.50 (1.25)	1.56 (1.30)	1.72 (1.43)	1.54 (1.28)	1.46 (1.22)	1.40 (1.17)	1.20
Ile	5.40 (1.13)	5.39 (1.12)	4.94 (1.03)	5.95 (1.24)	4.74 (0.99)	4.43 (0.92)	4.80
Leu	8.35 (1.03)	9.06 (1.12)	9.27 (1.15)	9.97 (1.23)	8.43 (1.04)	7.75 (0.96)	8.08
Lys	7.28 (0.93)	7.69 (0.98)	7.83 (1.00)	9.10^b^ (1.16)	6.85 (0.87)	6.48 (0.83)	7.84
Met	1.66 (1.04)	1.85 (1.16)	1.77 (1.11)	1.64 (1.03)	1.65 (1.03)	1.55 (0.97)	1.60
Phe	2.62 (1.06)	2.79 (1.13)	2.87 (1.16)	2.99 (1.21)	2.70 (1.09)	2.54 (1.02)	2.48
Thr	5.56 (1.04)	5.59 (1.04)	5.39 (1.01)	6.46 (1.21)	4.82 (0.90)	4.51 (0.84)	5.36
Trp	1.67 (1.39)	1.54 (1.28)	1.57 (1.31)	1.87 (1.56)	1.59 (1.33)	1.60 (1.33)	1.20
Val	4.80 (1.08)	5.00 (1.12)	4.83 (1.08)	4.46 (1.00)	4.54 (1.02)	4.29 (0.96)	4.46

Abbreviations: C‐RUSF, control ready‐to‐use supplementary food; WPC, whey protein concentrate.

a g/kg air dry weight.

b Based on reactive Lys determined using the guanidination method.

### 2.3 Setting and subjects

Children aged 6–59 months with MAM were enrolled at 27 rural feeding sites in southern Malawi from June 2018 to March 2019. The recruited children largely came from subsistence farming families whose diet consists predominantly of maize with very little animal protein. The harvest is typically once per year in April, and the incidence of malnutrition increases during the rainy season months of December through March, just prior to the annual harvest.

MAM was determined by mid‐upper arm circumference (MUAC) of greater than or equal to 11.5 cm and less than 12.5 cm or weight for height *z* score (WHZ) between −3 and −2, both without bipedal oedema. Children were excluded from the study if they had any congenital anomaly, cerebral palsy or a serious acute illness. They were also excluded if they were currently receiving or had received supplemental feeding in the past 6 months. The excluded children were fed supplementary food by the research team on a humanitarian basis, but their data were not collected or included in any study. Children with HIV and tuberculosis were included.

### 2.4 Study design

This was a randomized, double‐blinded, controlled clinical effectiveness trial in which participants received one of two foods, HiPro‐RUSF or C‐RUSF, and then were assessed for recovery from MAM. The primary outcome was recovery from MAM defined by achieving a MUAC greater than or equal to 12.5 cm or a WHZ greater than or equal to −2 within 12 weeks of treatment. If the child was enrolled on the basis of MUAC, then they were defined as recovered when their MUAC reached greater than or equal to 12.5 cm. Similarly, if they were enrolled on the basis of WHZ, they were defined as recovered when their WHZ reached greater than or equal to −2. If they were enrolled on the basis of both MUAC and WHZ, they were defined as recovered once they achieved either a MUAC greater than or equal to 12.5 cm or a WHZ greater than or equal to −2. For the children who did not recover from MAM, they were categorized as either developing SAM, death, remained MAM at 12 weeks or lost to follow‐up. Secondary outcomes were rates of weight, height, MUAC gain, time to graduation, treatment failure and adverse effects of the RUSF.

The study was powered to show at least a 3% difference in recovery from MAM between the groups with a 95% sensitivity and 80% power assuming the standard recovery rate is 80%, which has been seen in our previous feeding trials (Stobaugh et al., 2016).

The children were allocated to one of four colour groups, two colours randomized to HiPro‐RUSF and two randomized to C‐RUSF. Randomization was generated for the entire study using an online tool. Sets of opaque envelopes containing a colour corresponding to a feeding group were made according to the online randomizations. After consenting to participation, the caregivers picked an opaque envelope from a set that contained a colour corresponding to a feeding group. The packaging between food groups differed only in the colour of the label, which corresponded to the food group. Aside from the colour, the packaging was identical as was the taste and appearance of the food product. The research assistants, nurses, food distributers and participants were blinded to the groups. The food production workers were aware of the different groups but were not involved in the field work of enrolling, randomizing or collecting data.

### 2.5 Participation

Children were evaluated for MAM at 27 rural sites by senior nurses and research assistants. The weight was measured to the nearest 5 g after the child was placed in a seated scale (seca 334), and length was measured to the nearest millimetre on a rigid length board (seca 417). The MUAC was measured with a standardized insertion tape (TALC, St. Albans, UK) to the nearest millimetre. Children were evaluated for kwashiorkor by assessing for bilateral pitting oedema.

If the children met enrolment criteria, the senior nurses explained the study to the caregivers in the local language and consented them for participation both via verbal consent and written consent. The children were then randomized to the feeding groups. After randomization, the caregiver was interviewed for demographic information, appetite, recent infectious symptoms such as fever, cough, diarrhoea and for recent antibiotic use. The caregiver completed the Household Food Insecurity Access Scale (HFIAS) to assess household access to food (Coates, Swindale, & Bilinsky, 2007).

If two siblings were enrolled, they were given the same food to ensure that each child would receive the full ration of the properly randomized group. If the malnourished child had a healthy twin, the caregiver was given enough RUSF to feed each twin to limit sharing a single allotment between the twins. The goal of these interventions was to ensure that the malnourished child received a full dose of RUSF each fortnight.

A 2‐week weight‐based supply of either HiPro‐RUSF or C‐RUSF was distributed in 100‐g sachets at a dosage of 75 kcal/kg/day on the basis of randomization. After the caregivers received the RUSF, they were instructed on how to properly feed the RUSF to the enrolled child only, along with additional complementary foods, such as a local porridge. The caregiver was instructed to return with the child in a fortnight for repeat assessment. If the child remained with MAM upon return, the caregiver was given another 2‐week weight‐based ration of RUSF. However, if on a return visit the child had recovered from MAM, the child was graduated from the study. On this final visit, the caregiver was counselled on continuing the additional complimentary foods, such as the local porridge, and then received a small amount of cash compensation for their participation. If the child developed SAM on a return visit, the child was treated as an outpatient with ready‐to‐use therapeutic foods (RUTFs) or, if needed, was transported to the local hospital for inpatient care.

After commencing the clinical trial, the opportunity arose to measure the DIAAS in a pig model. Eight pigs had a T‐canula installed in the distal ileum to allow for sampling of ileal digesta. Pigs were then fed the C‐RUSF, the HiPro‐RUSF or a nitrogen‐free diet for 7 days. The initial 5 days were considered the adaptation period to the diet, but ileal digesta were collected on Days 6 and 7 for 9 h per day. Collected digesta were immediately stored at −20°C and later lyophilized and analysed. The ileal digestibility was calculated for the C‐RUSF and the HiPro‐RUSF and was then corrected for endogenous losses of AAs by using the data from the nitrogen free diet.

### 2.6 Data analysis

Data were tabulated and double entered into a database (Microsoft Access). The data were then cleaned by sorting for inconsistent values between the databases and resolving the discrepancy from the original data card. The data were also sorted to detect erroneous values, which were then investigated from the original data card.

Intention to treat analyses was used to compare categorical outcomes. The primary outcome, recovery from MAM, was analysed using Fisher's exact test (R, R Foundation for Statistical Computing 2013). The secondary outcomes—time to recovery, rate of weight gain at 2 weeks and at outcome and rate of MUAC gain at outcome—were calculated using the Student's *t* test. *p* values less than 0.05 were considered to be significant.

A logistic regression was performed including variables of enrolment MUAC, enrolment height for age z score (HAZ), age, sex, mother alive, father alive, father in home, child breastfed, number of siblings, number of siblings deceased and HFIAS score to identify potential risk factors for failure to recover from MAM. MUAC and HAZ were included in the model because they were not correlated using bivariate testing, whereas WHZ and weight for age z score were correlated with all other anthropometric indices.

### 2.7 Ethical considerations

The trial was registered at clinicaltrials.gov as NCT03549156 and was approved by the University of Malawi's College of Medicine Research and Ethics committee as well as the Human Research Protection Office at Washington University in St. Louis. The Malawian District Health Officer or District Nutritionist was notified and provided permission to carry out the study for each study site. Informed consent was obtained from the primary caretaker of the participant and documented by the caretaker's signature or thumbprint.

## 3 RESULTS

A total of 860 children were enrolled in the HiPro‐RUSF group and 877 children in the C‐RUSF group from June 2018 until March 2019 (Figure S1). Similar demographic, anthropometric and social characteristics were seen in the groups at enrolment, as one would expect with randomization (Table 5).

**TABLE 5:** Enrolment characteristics of children^a^

	Protein quality‐optimized RUSF *n* = 860	Control RUSF *n* = 877
Age (months)	16.7 ± 10.77	16.1 ± 9.89
Under 2 years	698 (81%)	720 (82%)
Sex (F)	517 (60%)	533 (61%)
Enrolment MUAC (mm)	12.2 ± 0.42	12.2 ± 0.43
Enrolment weight (kg)	7.28 ± 1.5	7.22 ± 1.4
Enrolment height (cm)	70.9 ± 8.7	70.6 ± 8.2
Enrolment WHZ	−1.68 ± 0.8	−1.65 ± 0.78
Enrolment HAZ	−2.71 ± 1.3	−2.71 ± 1.37
Enrolment WAZ	−2.75 ± 0.77	−2.73 ± 0.8
Mother deceased	10 (1%)	18 (2%)
Father deceased	10 (1%)	22 (3%)
Father in home	677 (79%)	694 (79%)
Child breastfed	621 (72%)	650 (74%)
Number of siblings	2.09 ± 1.9	2.11 ± 1.89
Number of siblings deceased	0.30 ± 0.13	0.35 ± 0.13
HFIAS		
• Food secure	8 (1%)	5 (1%)
• Mildly food insecure	7 (1%)	9 (1%)
• Moderately food insecure	115 (14%)	114 (13%)
• Severely food insecure	717 (85%)	741 (85%)

Abbreviations: HAZ, height for age z score; HFIAS, Household Food Insecurity Access Scale; MUAC, mid‐upper arm circumference; RUSF, ready‐to‐use supplementary food; WAZ, weight for age z score; WHZ, weight for height z score.

a Values are mean ± S.D. or number (percentage).

**FIGURE 1:**
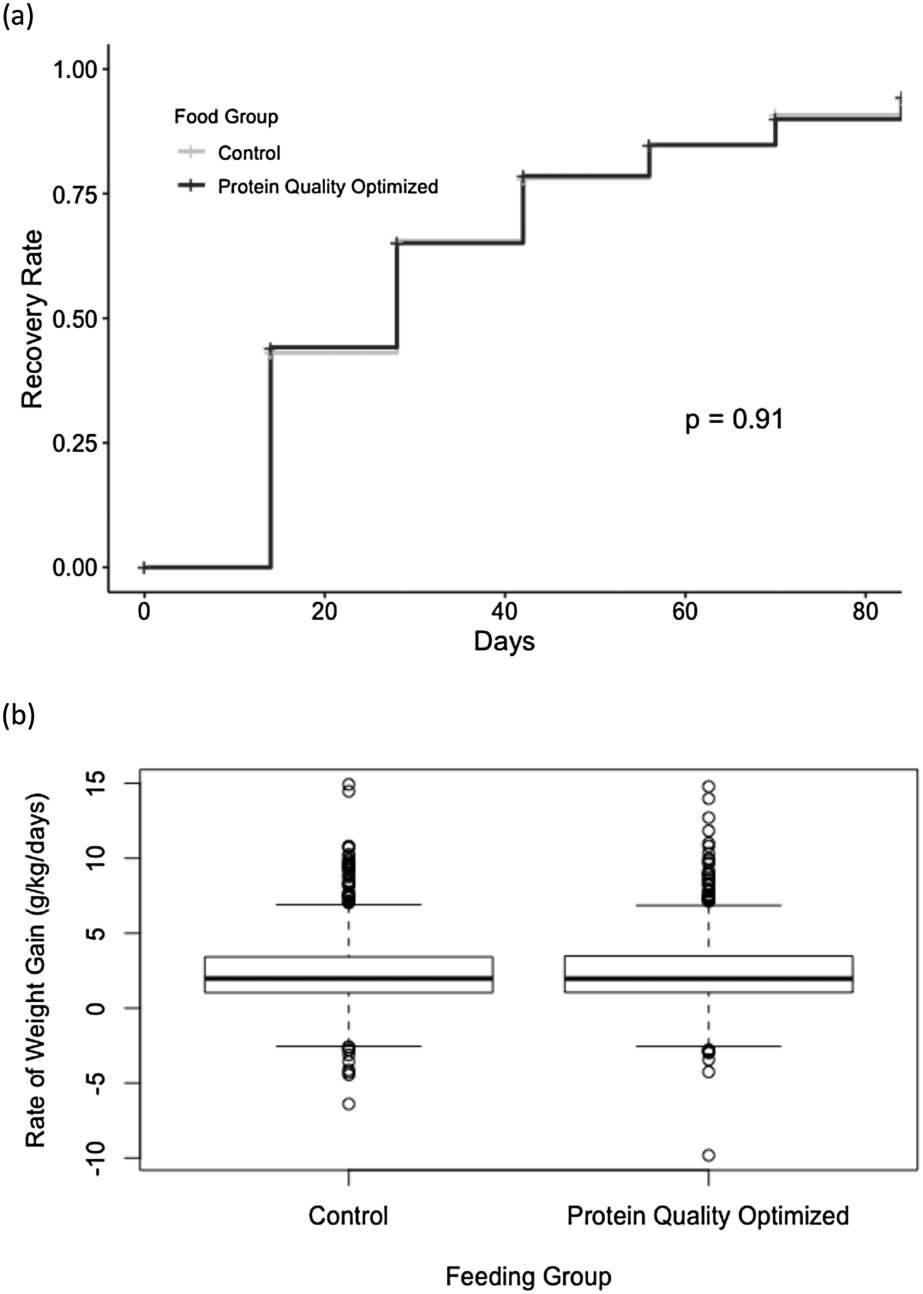
Recovery and weight gain in children with moderate acute malnutrition (MAM) receiving either protein quality‐optimized or control ready‐to‐use supplementary food (RUSF). (a) Time‐event plot of recovery from MAM by RUSF group and (b) rate of weight gain by food group. Boxplot presents median value as dark line, interquartile range as the box, minimum and maximum values as the whiskers and outliers are represented as dots (*p* = 0.78)

Among children receiving HiPro‐RUSF and C‐RUSF, 759/860 (88%) and 766/877 (87%) recovered, respectively (difference 1%, 95% CI, −2.1 to 4.1, *p* = 0.61, Figure 1, Table 6).

**TABLE 6:** Primary and secondary outcomes calculated using Fisher's exact test for categorical variables and Student's *t* test for continuous variables^a^

	Protein quality‐optimized RUSF *n* = 860	Control RUSF *n* = 877	Difference (95% CI)	*p* value
Recovery from MAM	759 (88%)	766 (87%)	1% (2.1% to 4.1%)	0.61
Develop SAM	39 (4.5%)	45 (5.1%)	−0.6% (−2.6% to 1.4%)	0.58
Remain MAM at 12 weeks	38 (4.4%)	40 (4.6%)	−0.2% (−2.15% to 1.7%)	0.91
Lost to follow‐up	24 (2.8%)	26 (3.0%)	−0.2% (−1.8% to 1.4%)	0.89
Rate of weight gain at 2 weeks (g/kg/day)	2.22 ± 2.8	2.26 ± 2.9	−0.04 (−0.23 to 0.31)	0.68
Rate of weight gain at outcome (g/kg/day)	2.44 ± 2.3	2.41 ± 2.5	−0.03 (−0.24 to 0.19)	0.82
Average time to recovery (days)	28.8 ± 19.4	28.6 ± 18.7	0.18 (−2.10 to 1.75)	0.86
Average weight gain (kg)	0.40 ± 0.3	0.40 ± 0.3	0.002 (−0.033 to 0.029)	0.90
Average MUAC gain (mm)	2.53 ± 3.0	2.47 ± 3.0	0.06 (−0.36 to 0.22)	0.64

Abbreviations: CI, confidence interval; MAM, moderate acute malnutrition; RUSF, ready‐to‐use supplementary food; SAM, severe acute malnutrition.

a Values are mean ± SD or number (percentage).

No significant adverse events attributable to the RUSFs were found.

The average time to recovery was 28.8 ± 19.4 days for the HiPro‐RUSF and 28.6 ± 18.7 days for the C‐RUSF (*p* = 0.86, Figure 1a). The rate of weight gain over the duration of feeding was 2.44 ± 2.31 g/kg/day for HiPro‐RUSF and 2.41 ± 2.48 g/kg/day for C‐RUSF (*p* = 0.82, Figure 1b, Table 6).

No variables apart from the enrolment MUAC and HAZ were found to be risk factors for failure to recover from MAM (Table S2).

Analysis of the study foods with the porcine model revealed a measured DIAAS of 82% for the HiPro‐RUSF with sulphur AAs being the limiting AA and 96% for C‐RUSF with histidine being the limiting AA (Table 7). The children in this study continued to eat their regular maize‐based diet. Taking into account the AA contributions of maize allows evaluation of the limiting AA of the child's total intake, not just that of the supplementary foods. When AAs of 125 g of maize are included, the DIAAS for the HiPro‐RUSF was 66% and for the C‐RUSF was 73%; lysine was the limiting AA for both diets (Table 7).

**TABLE 7:** DIAAS for each amino acid (mg/g of each AA divided by reference pattern for catch‐up growth in Table 1)

Essential amino acid	HiPro‐RUSF calculated	C‐RUSF calculated	HiPro‐RUSF measured	C‐RUSF Measured	HiPro‐RUSF measured + maize	C‐RUSF measured + maize
Histidine	1.02	0.63	1.02	0.96	0.95	0.91
Isoleucine	1.18	1.24	1.28	1.80	0.97	1.19
Leucine	1.09	0.99	1.10	1.38	1.08	1.22
Lysine	0.92^a^	0.96	0.98	1.15	0.66	0.73
Threonine	0.95	1.19	0.95	1.54	0.76	1.03
Valine	1.03	0.89	1.13	1.30	0.91	0.98
Sulphur AA	0.92^a^	1.17	0.82	1.25	0.90	1.12
Aromatic AA	1.22	1.12	1.33	1.30	1.08	1.05
Tryptophan	1.07	1.12	1.21	1.72	0.82	1.02

Abbreviations: AA, amino acid; C‐RUSF, control RUSF; DIAAS, digestible indispensable amino acid score; HiPro‐RUSF, protein quality‐optimized RUSF; RUSF, ready‐to‐use supplementary food.

a Lysine was 0.921 and SAA was 0.923, so lysine was the limiting AA. However, in the pig data, when we use the new reference pattern for catch‐up growth, the SAA is now the least abundant/digestible AA.

## 4 DISCUSSION

This study showed similar, high rates of recovery from MAM in children receiving either HiPro‐RUSF or C‐RUSF. Likewise, the rates of weight gain, average weight and MUAC gain, and time to recovery did not differ between the HiPro‐RUSF and C‐RUSF groups. Calculation of DIAAS in the study foods and measurement of DIAAS in an intricate animal model varied considerably. For the calculated DIAAS, HiPro‐RUSF was 92% and C‐RUSF was 63%; for the measured DIAAS, HiPro‐RUSF was 82% and C‐RUSF was 96%. If DIAAS in the supplementary food was a predictor of growth in recovery from MAM, we would not expect growth rates to be as similar as we observed. If the measured DIAAS in RUSF was a determinant of growth, the C‐RUSF would have been superior. Thus, our hypothesis that protein quality is a characteristic of supplementary food that determines growth is not supported by these data.

With highly nutritious RUSF, we achieved recovery rates close to 90%, in contrast to other studies in sub‐Saharan Africa where more typically 70%–80% recovery has been demonstrated for supplementary feeding RUSF in MAM (Stobaugh et al., 2016; Suri et al., 2016). A 2017 systematic review found an overall recovery rate of 81% over eight randomized controlled trials using various RUSF formulation for the treatment of MAM (Gera, Pena‐Rosas, Boy‐Mena, & Sachdev, 2017).

An accurate metric to compare diverse protein sources in supplementary food products that corresponds to physiologic requirements in recipients is needed by the agencies that regulate supplementary food product composition. Protein quality has been suggested by some experts as such a measure. Our RUSFs had similar types of dietary protein delivered in foods with protein quality scores that differed by 14% (Table 7). Both RUSFs in this study had a high proportion of dairy protein, which has been shown in previous studies to associate with improved anthropometric scores (Schlossman et al., 2017) and better recovery from MAM (Stobaugh et al., 2016). It had previously been hypothesized that the higher protein quality of dairy protein when compared with vegetable protein could account for the improved recovery from MAM. However, the controlled conditions of this trial suggest that in supplementary food products for MAM, protein quality metrics do not affect clinical effectiveness.

### 4.1 Protein quality metrics

The DIAAS and PDCAAS scores were calculated on the basis of the least abundant and digestible AA in standard references. In the calculations, the limiting AA for the C‐RUSF was histidine, whereas for the HiPro‐RUSF, it was lysine. However, as the excellent PDCAAS and DIAAS scores in the HiPro‐RUSF indicate, there were adequate amounts of digestible lysine per our calculations. Measurement of the actual DIAAS score yielded a score of 82% for the HiPro‐RUSF and 96% for C‐RUSF. In fact, in the measured ileal sampling of these supplementary foods, the C‐RUSF DIAAS was higher than the HiPro‐RUSF. When the AA contributions of maize, the staple crop in this population, was included in the analysis, the DIAAS of the HiPro‐RUSF and C‐RUSF were similar (66% and 73%, respectively, Table 7).

Our study calculated C‐RUSF and HiPro‐RUSF protein quality scores on the basis of available data of AA content and digestibility for the ingredients used. However, there is significant variation in WPC (Table 4) that can only be accounted for by measuring the specific WPC that was used to produce the RUSF. The variability of the AA content in WPC suggests that calculated protein quality values may be inaccurate, as was the case for C‐RUSF. This uncertainty may limit the use of WPC in foods where protein quality is specified.

The calculations that we made for protein quality of our C‐RUSF and HiPro‐RUSF did not include information on the staple diet that is eaten in rural Malawi. As this was a supplementary feeding programme, the caregivers were encouraged to continue to feed the children their regular meals, which is a corn‐based diet. Maize protein is limited in tryptophan and lysine but sufficient in all other essential AAs. Taking into account the full nutrient intake instead of just the contribution from the RUSF may change the determination of the limiting AA and the overall performance of the RUSF. For example, though C‐RUSF had lower calculated protein quality scores, its limiting AA was histidine, which maize provides. In contrast, the HiPro‐RUSF, though it had a higher calculated protein quality score, was limiting in lysine, which is also limiting in maize. Therefore the C‐RUSF might have performed similarly to HiPro‐RUSF in this study because of the contribution of the rest of the child's diet. This is why protein overall quality scores should not be relied upon, but rather each AA should be taken as an individual nutrient.

It is likely that the protein quality scores did not correlate with clinical outcomes because though the scores are based on the least abundant AA, not all AAs are equal in their clinical relevance to nutrition and recovery from MAM. For example, being insufficient in histidine, such as in our C‐RUSF, may not be as clinically important for recovery from MAM as being insufficient in another AA but would give the same protein quality score.

Histidine has ambiguous information on whether it is, in fact, an essential AA. There is no difference in nitrogen balance in people on short‐term histidine‐deficient diets and only equivocal data for those on histidine‐deficient diets for a month or more (Joint WHO/FAO/UNU Expert Consultation, 2007). There are large stores of histidine in both haemoglobin in blood and carnosine in skeletal muscle, which can be broken down to release histidine in times of deficiency. These abundant reservoirs make it difficult to study if histidine is an essential AA, as most studies are shorter than the time period necessary to deplete histidine stores. Infants lack sufficient amounts of carnosinase, the enzyme to breakdown carnosine, to allow histidine release, so histidine is considered an indispensable AA in international guidelines despite the equivocal evidence (Joint WHO/FAO/UNU Expert Consultation, 2007).

In contrast, other essential AAs appear to be more important to growth. Certain indispensable AAs serve as signalling molecules in metabolic pathways that stimulate anabolism in addition to their role in protein synthesis. Leucine directly stimulates muscle protein synthesis via the rapamycin signal cascade, and branched chain AAs (isoleucine, valine) act as substrates for gluconeogenesis (Millward, Layman, Tomé, & Schaafsma, 2008). Historically, lysine, methionine and tryptophan were seen as the most limiting AAs in poor‐quality proteins and thus the most important to supplement (Joint WHO/FAO/UNU Expert Consultation, 2007; Millward et al., 2008). However, the relative importance of each essential AA is not considered in the DIAAS and PDCAAS scores. In considering treatment foods for MAM, it may well be more relevant to view the essential AAs as separate nutrients that cannot be aggregated into an overall protein quality score.

### 4.2 Limitations

Limitations of this study are that it was conducted in rural southern Africa, where maize is the staple food and gut inflammation is ubiquitous. Our findings may not apply to other populations consuming different staple foods. Another limitation of any dietary protein quality scoring system is that the gut microbiota's contribution to essential AA provision is unaccounted for. Both of the RUSFs used in this trial provided large amounts of the prebiotic lactose, which may generously feed the microbiota and facilitate provision of essential AAs. Although the microbiota are versatile biosynthetic sources of nutrients, they cannot create nitrogen. The overall nitrogen content of the diets in malnourished individuals might be an important determinant of recovery. Previous studies have suggested that the colonic microbiome significantly recycles nitrogen found in ingested protein and that the rate of this recycling may increase when protein consumption is reduced (Jackson, Bundy, Hounslow, Murphy, & Wootton, 1999; Millward et al., 2000). One study gave labelled oral doses of nitrogen to malnourished infants and found significant recycling of nitrogen into the systemic circulation similar to other studies. More significantly, they found the labelled nitrogen in the infant's AAs, including essential AAs. The labelled nitrogen found in essential AAs could only have come from the faecal microbiota as essential AAs cannot be synthesized by humans (Millward et al., 2000). The study limited its AA analysis to histidine and lysine and was not designed to measure the true contribution of the intestinal microbiota to serum essential AAs; nonetheless, the authors estimated that about 58 mg of lysine/kg/day could be added by the faecal microbiota. On the basis of these findings, it is possible that the overall nitrogen content in the RUSF allows the colonic microbiota to synthesize additional essential AAs and thus could overcome the single limiting AA from which the protein quality scores DIAAS and PDCAAS are based. Although the microbiota as a source of essential AAs is not easily quantified, it should not be discounted.

The study population was rural African children aged 6–59 months who developed acute wasting in conjunction with household food insecurity. Extension of our findings to children from other demographics or children with chronic illnesses is not warranted. This was not a study comparing dairy protein with vegetable protein. Our data do not inform the nutrition community about the suitability of substitutions of dietary plant protein for dairy in supplementary food products. Looking ahead, we recommend further research into the effect of different protein sources on clinical outcomes when using supplementary food products. We encourage caution when building recommendations for supplementary food products around protein quality scores, given the lack of high‐quality clinical trial evidence to suggest their validity.

## 5 CONCLUSIONS

Protein quality scores of RUSF are ambiguous, contingent upon assumptions of ingredient AA content and not reflective of overall diet. In this study, we found that protein quality score of the RUSF does not correlate with clinical recovery from MAM. Our RUSFs provided the same amount of total protein content, and both had high amounts of dairy protein but differed in protein quality score when measured. However, both delivered excellent clinical outcomes, as seen in the high rate of recovery from MAM. Protein quality scores are flawed because they do not differentiate between the relative importance of different AAs in growth or the contributions of the colonic microbiota to essential AA. The needed AA contribution from RUSF also depends on the AA contribution from the supplemented foods because it is the AA composition of the total daily intake that determines the adequacy of an individual's daily AA intake. It is, therefore, unlikely that a defined DIAAS or PDCAAS score will optimize the AA composition of all RUSFs. On the basis of these findings, we would recommend against a specific protein quality being used to set guidelines to standardize RUSF.

## CONFLICTS OF INTEREST

The authors declare that they have no conflicts of interest.

## CONTRIBUTIONS

MCG and MM designed the study and developed the ready to use supplementary food recipes. MCG, RR, KK, JKB and MM were involved in the enrolment of subject and data collection. HHS measured the DIAAS with an animal model and measured AA content of various WPCs. RR analysed the data and wrote the initial draft of the manuscript. MJM had responsibility for the study's final content. All the coauthors reviewed the manuscript and contributed substantial edits for the final version.

## Supporting information

Table S1: Nutrient composition of intervention foods per 100 grams of finished product, compared with minimum specifications for ready‐to‐used supplementary food (WFP, 2019)
Table S2: Logistic Regression for Risk Factors for Treatment Failure
Table S3: Standardized ileal digestibility (SID) of amino acids (AA) in ingredients|
Figure S1: Study Flow Diagram
