# Supplementary material for: Protein quality in ready‐to‐use supplementary foods for moderate wasting
Source: Matern Child Nutr. 2020 May 19;16(4):e13019. doi: 10.1111/mcn.13019 (PMC7507576; doi:10.1111/mcn.13019)
Supplement: Supplementary file 1 — Table S1: Nutrient composition of intervention foods per 100 grams of finished product, compared with minimum specifications for ready‐to‐used supplementary food (WFP, 2019) Table S2: Logistic Regression for Risk Factors for Treatment Failure Table S3: Standardized ileal digestibility (SID) of amino acids (AA) in ingredients| Figure S1: Study Flow Diagram [file MCN-16-e13019-s001.docx]

Supplementary Appendix

Table S1: Nutrient composition of intervention foods per 100 grams of finished product, compared with minimum specifications for ready-to-used supplementary food (WFP, 2019)

|  | **Protein Quality Optimized RUSF** | **Control RUSF** | **WFP Recommended per 100g** |
| --- | --- | --- | --- |
| **Minerals** |  |  |  |
| Biotin (µg) | 60 | 60 | 65 |
| Calcium (mg) | 535 | 535 | 413 |
| Copper (mg) | 1.4 | 1.4 | 1.2 |
| Iodine (µg) | 100 | 100 | 110 |
| Iron (mg) | 10 | 10 | 10 |
| Magnesium (mg) | 150 | 150 | 100 |
| Manganese (mg) | 1.2 | 1.2 | 1.0 |
| Phosphorus (mg) | 450 | 450 | 319 |
| Potassium (mg) | 900 | 900 | 350 |
| Selenium (µg) | 20 | 20 | 15 |
| Zinc (mg) | 11 | 11 | 11 |
| **Vitamins** |  |  |  |
| Folic acid (µg) | 98.50 | 255.61 | 500 |
| Niacin (mg) | 13.0 | 13.0 | 16 |
| Pantothenic acid (mg) | 4.0 | 4.0 | 4.9 |
| Riboflavin (mg) | 2.1 | 2.1 | 2.6 |
| Thiamine (mg) | 1.0 | 1.0 | 1.5 |
| Vitamin A (RAE) (µg) | 1150 | 1150 | 1050 |
| Vitamin B-6 (mg) | 1.8 | 1.8 | 2.2 |
| Vitamin B-12 (µg) | 2.7 | 2.7 | 2.9 |
| Vitamin C (mg) | 60 | 60 | 90 |
| Vitamin D (µg) | 15 | 15 | 18 |
| Vitamin E (µg) | 16 | 16 | 20 |
| Vitamin K (µg) | 27 | 27 | 27 |

Table S2: Logistic Regression for Risk Factors for Treatment Failure

|  | **Estimate** | **Standard Error** | **P value** | **OR** | **2.5 %** | **97.5 %** |
| --- | --- | --- | --- | --- | --- | --- |
| Enrollment MUAC | 2.304 | 0.261 | <0.0001 | 10.016 | 6.003 | 16.71 |
| Enrollment HAZ | 0.176 | 0.62 | 0.005 | 1.193 | 1.055 | 1.348 |

Table S3: Standardized ileal digestibility (SID) of amino acids (AA) in ingredients

| Item, % | RUSF experimental | RUSF control |
| --- | --- | --- |
| Indispensable amino acids |  |  |
| Arg | 96.0 | 104.2 |
| His | 92.0 | 96.3 |
| Ile | 87.9 | 96.1 |
| Leu | 91.4 | 97.3 |
| Lys | 90.0 | 92.6 |
| Met | 92.7 | 94.7 |
| Phe | 92.6 | 97.0 |
| Thr | 85.8 | 89.7 |
| Trp | 87.4 | 97.8 |
| Val | 88.6 | 94.0 |

Figure S1: Study Flow Diagram

- 851 Children Completed the Study
  - 766 recovered from MAM
  - 40 remained MAM at 12 weeks
  - 45 developed SAM
- 26 Children Lost to Follow Up
- 121 excluded
  - 53 healthy twins
  - 42 enrollment error
  - 7 measurement errors
  - 2 completed study early
  - 17 did not meet age criteria

877 Children Randomized to Control RUSF

860 Children Randomized to High Protein RUSF

- 836 Children Completed the Study
  - 759 recovered from MAM
  - 38 remained MAM at 12 weeks
  - 39 developed SAM
- 24 Children Lost to Follow Up

1858 Children Provided Consent

1737 Children Randomized
